# Case of Opium Poisoning

**Published:** 1873-12

**Authors:** H. S. Schell


### Case of Opium-Poisoning—Use of Atropia.

By H. S. SCHELL, M. D.

At 7.30 P. M., November 8, 1873, I was called to see L. M., a young woman, who at six o'clock the same evening had taken one and a half fluidounces of laudanum for the purpose of destroying her life. I found her unconscious, her extremities cold, muscles relaxed; pulse 60, and full, but not strong; respiration snoring; face of a dusky hue; irides immobile, pupils about $\frac{1}{32}$ inch in diameter, and corneæ insensible.

I had brought with me a couple of yards of india-rubber tubing of small diameter, and a syringe, and, after passing one end of the tube into the stomach, and starting the fluid in motion through it by the syringe, I allowed the other end to fall into a basin by the bedside and thus act as a siphon. In this way a couple of ounces of brownish fluid, smelling somewhat of laudanum, were discharged, when the flow ceased, and could not be again started. While preparing to inject some warm water to wash out the stomach, the tube was accidentally drawn out of the œsophagus, and I was unable to re-introduce it. She had taken no food during the day, and the brownish fluid was evidently mostly beer, of which she had drunk a glass a short time before taking the laudanum. I thought it better, however, to give her an emetic of mustard and salt, to get rid of whatever laudanum might remain; but it was with the greatest difficulty she was sufficiently aroused to be induced to swallow it. I gave her at the same time thirty drops of the tincture of belladonna, and repeated the latter every fifteen minutes until after ten o'clock. The emetic, not acting, was repeated in twenty minutes, and in twenty minutes thereafter was followed by a scruple of sulphate of zinc, and the fauces were tickled with a feather; all without result.

At 9.30 the pulse was somewhat more frequent, but weaker; the condition of the respiration and temperature of the surface remained about the same; and I now noticed for the first time that the optic axes were considerably divergent, the eyeballs being also rolled somewhat upward.

At 10.15, after rousing her to give her a dose of tincture of belladonna, there was an almost convulsive struggle of resistance, attended by violent excitement, and followed by sudden and complete collapse. There was an entire cessation of respiration, the pulse was barely perceptible, the face pale, lips livid, pupils unchanged, and eyes rolled up and outward to the extreme limits of their orbits.

As shaking, slapping, the cold douche, etc., failed to re-excite the act of breathing, I commenced artificial respiration by Marshall Hall's method; and, after keeping it up for ten minutes without improvement, I instructed an assistant how to continue it, and returned to my office for my hypodermic syringe, at the same time sending for a solution of the sulphate of atropia, gr. j ad f℥j. On my return, ten minutes afterwards, I found her in the same condition as when I left; not an inspiration being drawn except by the artificial method, and only the feeble fluttering of the heart to show that she was still living.

I at once, however, injected $\frac{1}{48}$ grain of atropia under the skin of the arm, and in a short time had the satisfaction of seeing a natural act of inspiration, and of finding a decided pulse again under my fingers, beating at the rate of 108 in the minute. The pupils soon dilated to $\frac{1}{16}$ inch, and she vomited five or six ounces of fluid. Artificial respiration was of course discontinued, but in a short time the pulse fell again to 90, alarming symptoms reappeared, and twenty minutes after the first injection of atropia I repeated it. The pulse soon rose again. There was copious emesis, but no odor of opium about the ejected fluid. She asked for water, which was given freely, and in the half-unconscious intervals of vomiting she kept up a pretty general scratching of the surface of the body, and rubbing of the nose, showing that she was beginning to be annoyed by the action of the opium upon the skin.

In a little while, however, there was a diminution of the pulse-rate, return of coma, etc., and in half an hour from the last injection I repeated the atropia. The effect now seemed to be more permanent; the pulse rose to 110 and became stronger, the pupils dilated to a little over $\frac{1}{8}$ inch in diameter, and remained so, responding, however, to the action of light. She complained of seeing objects double, and was very tremulous.

When I left her, after 1 A.M., she was dozing quietly, could be easily aroused, her respiration was full, skin warm, pulse 100 and moderately strong, and she had still some divergence of the optic axes.

When I saw her the next morning at 10 o'clock she was sitting by the stove, apparently quite well: the derangement of vision had disappeared, but the thickly-coated tongue and occasional nausea were evidences of considerable derangement of the stomach.

I learned that she had had abundant diuresis during the night; but, owing to accidental circumstances, I was unable to procure a specimen of the urine for examination.

The principal point of interest in connection with this case is the striking manner in which it illustrates the stimulant effect of atropia in moderate doses upon the par vagum.

As to its action upon the gastric branches, it will be remembered that the stomach first responded to the action of the emetics about two hours after they were taken, and in a few minutes after the first hypodermic injection, and freely after the second one.

Tincture of belladonna had been previously given by the mouth, and about f℥ss had been used from the bottle containing it; but, in consequence of the difficulties attending the administration, it is probable that not more than fʒiij were actually swallowed. Owing, however, to the lethargic condition of the stomach, produced by the opium, I imagine that but little of the three fluidrachms was absorbed, and that the most of it was afterwards vomited.

In the case of a man who had taken thirty grains of opium and ten or twelve ounces of whisky, reported by Dr. McGee,*—the use of various emetics for a couple of hours produced no result; but, after the subcutaneous injection of $\frac{1}{4}$ grain of atropia, free vomiting took place immediately.

In Dr. Walker's case, † a girl fifteen years old had taken six or eight grains of opium, and emetics were used ineffectually until after the hypodermic injection of $1\frac{1}{2}$ grains ext. belladonna.

In Dr. Todd's case, ‡ of a man of intemperate habits, who had taken tr. opii fʒx, emetics failed to excite vomiting. Atropia was administered subcutaneously at 10.30 P.M., but there was no emesis until after he had been violently rolled about the bed at 3 A.M. In this instance the stomach was probobly unusually callous to impressions, in consequence of the habits of the individual.

The action of the remedy upon the cardiac branches of the pneumogastric is seen in the manner in which the pulse rose and strengthened after each use of the hypodermic syringe.

As to the lungs, it has been seen that the use of artificial respira-

* Am. Jour. Med. Sci., p. 282, July, 1869.

† Am. Jour. Med. Sci., p. 282, Jan., 1872.

‡ Am. Jour. Med. Sci., p. 131, Jan., 1873.

tion continued for twenty-five or thirty minutes produced in that time no action of the respiratory muscles, but that the breathing became regular and natural soon after the use of atropia.

I should conclude, therefore, that when the patient is in a comatose condition when first seen, it would be better to administer $\frac{1}{60}$ to $\frac{1}{30}$ grain of atropia subcutaneously at once, and give an emetic as quickly as possible thereafter; and it seems probable that the action of the emetic may be aided by rolling the patient. If, on the contrary, the sulphate of zinc or mustard is allowed to remain in the stomach for an hour or two, it sets up a degree of irritation, or even inflammation, which requires special treatment for its relief and often lasts for several days.

It would seem, too, that there is no advantage in pushing the atropia, until the pupils are widely dilated, but that it is sufficient to give enough to stimulate the nervous centres whence the pneumogastric springs to the degree of action necessary to keep the heart and lungs in motion until the system can rid itself of the poison.

A peculiar feature of this case was the divergence of the optic axes,—a symptom which I do not remember to have seen noticed elsewhere, but which was probably the cause of the diplopia mentioned in the cases recorded by Dr. Johnson* and Dr. Wood, † as it evidently was in this instance. It is possible that there may have been sufficient tincture of belladonna absorbed by the stomach to act upon the nervous centres in the floor of the fourth ventricle, whence the abducens and vagus take their deep origin, and that all the other muscles of the eye, being temporarily paralyzed by the opium, were powerless to resist the external rectus.—*Phil. Med. Times.*

* Medical Times and Gazette, Feb. 15, 1873.
† American Jour. Med. Science, April, 1873, p. 342.

